# Induction, rapid fixation and retention of mutations in vegetatively propagated banana

**DOI:** 10.1111/j.1467-7652.2012.00733.x

**Published:** 2012-12

**Authors:** Joanna Jankowicz-Cieslak, Owen A Huynh, Marta Brozynska, Joy Nakitandwe, Bradley J Till

**Affiliations:** Plant Breeding and Genetics Laboratory, Joint FAO/IAEA Division of Nuclear Techniques in Food and Agriculture, IAEA Laboratories Seibersdorf, International Atomic Energy Agency, Vienna International CentreVienna, Austria

**Keywords:** Musa, induced mutations, TILLING, enzymatic mismatch cleavage, chimerism, ethyl methanesulphonate

## Abstract

Mutation discovery technologies have enabled the development of reverse genetics for many plant species and allowed sophisticated evaluation of the consequences of mutagenesis. Such methods are relatively straightforward for seed-propagated plants. To develop a platform suitable for vegetatively propagated species, we treated isolated banana shoot apical meristems with the chemical mutagen ethyl methanesulphonate, recovered plantlets and screened for induced mutations. A high density of GC-AT transition mutations were recovered, similar to that reported in seed-propagated polyploids. Through analysis of the inheritance of mutations, we observed that genotypically heterogeneous stem cells resulting from mutagenic treatment are rapidly sorted to fix a single genotype in the meristem. Further, mutant genotypes are stably inherited in subsequent generations. Evaluation of natural nucleotide variation showed the accumulation of potentially deleterious heterozygous alleles, suggesting that mutation induction may uncover recessive traits. This work therefore provides genotypic insights into the fate of totipotent cells after mutagenesis and suggests rapid approaches for mutation-based functional genomics and improvement of vegetatively propagated crops.

## Introduction

Asexual or vegetative propagation is common to many plant species owing to pools of totipotent stem cells maintained by plants (Murashig, 1974; Priestley, 1929). Examples include apple, banana, cassava, citrus, coffee, grapevine, hops and potato and thus represent major economic and food security crops. Vegetatively propagated plants can be further defined as either facultative, whereby sexual or meiotic propagation is possible but sometimes inefficient, or obligate where mitotic propagation is the only means available (van Harten, 1998). Prolonged or continual mitotic propagation can alter the genetic make-up of organisms. Lack of meiosis, recombination and independent assortment provide a means of accumulating spontaneous deleterious mutations that cannot be expunged efficiently from a population, a phenomenon known as Muller’s ratchet (Muller, 1932). This has led to models for the preponderance of sexual propagation in extant species (Felsenstein, 1974; Muller, 1964). For example, lack of meiotic propagation means that new genotypic combinations cannot be easily created. This has major implications for crop improvement as crossing is a key feature of most plant improvement schemes.

One strategy for inducing novel genetic variation in both sexually and asexually propagated species is mutagenesis. Since the pioneering work of Muller and Stadler, mutagenesis has been established as an efficient tool for forward genetics, plant breeding and more recently reverse genetic approaches (Ahloowalia *et al.*, 2004; Henikoff *et al.*, 2004; Muller, 1927; Stadler, 1928). In the past decade, high-throughput mutation discovery methods have been developed that allow measurements of densities and spectra of induced mutations without the need for an expressed phenotype. Thus, a more accurate assessment of the total effect of mutagenic treatment can be made. To date, the largest data sets come from chemical mutagenesis of plants and animals developed for TILLING (Targeting Induced Local Lesions IN Genomes) screens. TILLING is a strategy that combines random mutagenesis with mutation discovery for a reverse genetic approach that enables targeted recovery of a spectrum of point mutations in any gene in a genome (Kurowska *et al.*, 2011; McCallum *et al.*, 2000). Widely applicable, TILLING has been established for a range of plant and animal species. In crops, it can be a nontransgenic alternative to create novel genetic diversity and has been used for the improvement of species such as wheat and potato, where altering starch quality is of commercial importance (Muth *et al.*, 2008; Slade *et al.*, 2005). The most commonly reported mutagen, ethyl methanesulphonate (EMS), causes primarily GC-AT base pair transition mutations in many species including *Arabidopsis thaliana*, *Triticum durum* (tetraploid wheat), *Triticum aestivum* (hexaploid wheat), *Zea mays* and *Caenorhabditis elegans* (Gilchrist *et al.*, 2006; Kurowska *et al.*, 2011). In other species, such as *Drosophila melanogaster*, *Hordeum vulgare* and *Oryza sativa*, over 25% non-GC-AT changes have been reported (Caldwell *et al.*, 2004; Cooper *et al.*, 2008; Till *et al.*, 2007). This divergence from transition changes may be an artefact intrinsic to the way populations were produced or maintained, or may represent differences in mismatch repair unique to particular species or genotypes. Regardless of species or genotype, nearly all reported mutations have been single nucleotide changes. While the majority of induced point mutations are predicted to be functionally silent, other nucleotide changes such as non-sense, mis-sense, RNA splicing defects and regulatory alterations result from chemical mutagenesis. Such changes can have varying effects on gene expression and protein function (Barkley and Wang, 2008; Vaddepalli *et al.*, 2011). Reported densities of induced mutations in sexually propagated species range from 1 mutation per 1 million base pairs (Mb) to 1 in 25 kilobase pairs (kb) (Jankowicz-Cieslak *et al.*, 2011). While densities can be affected by treatment regime and variations in mutation repair pathways, a trend can be observed where higher densities are achieved with increasing ploidy, a phenomenon first observed phenotypically in wheat (Stadler, 1929). An optimal mutation density allows for smaller population sizes and more rapid recovery of desired alleles and therefore is a key to successful breeding and functional genomics approaches.

Little is known with regard to the achievable spectrum and density of mutations in asexually propagated species. Confounding factors include previous reliance on phenotypic observations that can be influenced by environmental conditions and epigenetic variation, the reliance on dominant or hemizygous alleles in the first generation (M_1_) and the presence of genotypic heterogeneity or chimerism after mutagenesis. Such heterogeneity occurs because at the time of mutagenesis, it is expected that all cells accumulate different EMS-induced mutations randomly throughout the nuclear genome. This is problematic because measured phenotypes and genotypes may represent only a subset of the variation in the material and may not be inherited. Chimerism can be eliminated in sexually propagated crops through the single cell–based mechanisms of meiosis and sexual reproduction (Comai and Henikoff, 2006). While straightforward, this requires the production of a M_2_ population which necessitates additional resources and time. Further, a structured population typically following single seed descent is often employed to ensure that induced alleles are sampled only once as oversampling increases time and costs and confounds estimations of mutation density. For vegetatively propagated crops, strategies have been developed that employ successive rounds of tissue isolation and bisection aimed at reducing the genotypic complexity of the resulting plantlets (van Harten, 1998). However, methods for rapid dissolution of genotypic heterogeneity, or chimerism, in mitotically propagated tissues have yet to be evaluated at the genomic level.

The genus Musa, consisting of bananas and plantains, represents the fourth most important food crop in some of the world’s least developed countries. Edible varieties tend to be triploid and parthenocarpic. They are seedless and thus must be propagated vegetatively. Although banana is a major export commodity worth over 28 billion US dollars annually, 87% of Musa production is consumed locally (http://faostat.fao.org/site/339/default.aspx 2009 accessed 21 February 2012). Plantations consist of cloned plants that are nearly genetically identical and uniform, which makes the crop particularly susceptible to fungal pathogens causing biotic diseases such as panama and black sigatoka. This has led to speculation that worldwide banana production may cease to be sustainable in the near future, threatening economic stability and food security in developing countries in the tropics and subtropics (Heslop-Harrison and Schwarzacher, 2007). Therefore, we sought to develop a platform for the induction of stably heritable point mutations in mitotically propagated bananas. We observe rapid dissolution of chimeric sectors in EMS-treated meristematic tissues and discuss models for this unexpected result. The findings have potential practical implications for mutagenesis-based functional genomics and breeding of many other vegetatively propagated plants.

## Results

### Development of an EMS-mutagenized population of vegetatively propagated banana

To establish optimal concentration ranges for EMS mutagenesis of banana shoot tips, cultures were treated with 4 EMS concentrations (0.25%, 0.5%, 1% and 1.5%) and 2 incubation times (2 and 4 h). A total of 25 cuttings were evaluated for survival rate and fresh weight per treatment regime. Regardless of concentration of EMS, all explants incubated for 2 h survived the treatment. After 4 h incubation, only the 1.5% EMS treatment was lethal to the *in vitro* shoot tip cuttings. The percentage reduction in fresh weight of *in vitro* plantlets was used to choose mutagenesis conditions for the development of a large population suitable for mutation discovery, evaluation of mutation inheritance and measuring the rate at which plants become genotypically homogeneous. A target range weight reduction of 40%–50% was selected to balance the need for sufficient plant growth while maximizing the density of induced mutations.

For bulk mutagenesis, 4000 *in vitro* shoot tip cuttings of the triploid ‘Grande Naine’ variety were prepared. Owing to the potentially confounding variables of EMS absorbance time and possible cytotoxic effects on cell function, four different mutagenic treatments were selected to achieve the weight reduction ranges described above. Treatment batches consisted of 1000 banana shoot tips. Mutagenesis was performed at room temperature for 3 h (1% EMS), 6 h (0.5% EMS), 24 h (0.125% EMS) and 48 h (0.06% EMS), respectively. Survival rates ranged from 52% (24 h) to 87% (48 h) with no trend observable between survivability and either EMS dosage or time (see Table S1). A total of 3004 mutagenized samples survived the treatments.

**Figure 1:**
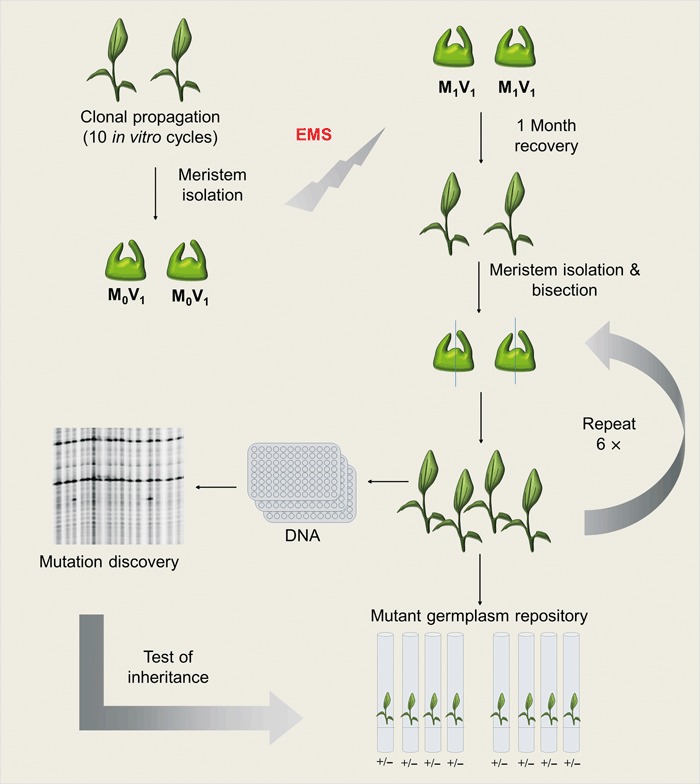
A strategy for meristematic mutagenesis, chimera dissolution and mutation recovery in vegetatively propagated banana. Clonally propagated isogenic plants are prepared through 10 rounds of *in vitro* multiplication. Shoot apical meristems are isolated and soaked in EMS, then allowed to recover for 1 month. Individuals from the first vegetative generation (M_1_V_1_) are propagated through meristem isolation and longitudinal bisection to produce the M_1_V_2_ generation. Successive rounds of isolation and division are performed to reduce genotypic heterogeneity, and the number of individuals approximately doubles each generation. Tissue is collected from M_1_V_6_ individuals, and DNA is extracted and screened for induced mutations. The inheritance of isolated mutations is evaluated in the M_1_V_6_ and subsequent generations, allowing for estimations of the rate of chimera dissolution.

To reduce the chance of chimeric sectors in mutagenized plants, meristems were repeatedly isolated and longitudinally bisected (Figure 1). This results in a reduction in the number and genotypic diversity of meristematic cells that divide to produce progeny. Plants were mitotically propagated through six vegetative cycles to the M_1_V_6_ generation. Little is known about the mechanism of chimera dissolution in banana and other vegetatively propagated plants. A previous study in banana relying on the measurement of cytochimeras looking at polyploidy induced by the treatment of plants with colchicine suggested that chimeras are at least partially removed by the third or fourth generation, and so the expectation is that all EMS-mutagenized plants at the M_1_V_6_ generation will be genotypically homogenous (Jain *et al.*, 2011; Roux *et al.*, 2001). Seven hundred and sixty-eight plants were chosen at random for the evaluation of mutation density and spectrum (see Table S2). A total of 23 primer pairs specific for putative open reading frames were designed from BAC sequence to amplify gene fragments between ∼755 and 1500 bp. Forty-eight per cent (11/23) of primer pairs produced both high-quality TILLING data and Sanger sequencing reads and were included in this study (Table 1). The remaining primers produced poor-quality Sanger sequencing reads, potentially owing to high levels of natural background polymorphisms driving primer mispriming (Till *et al.*, 2010). Samples were subjected to TILLING screens using the validated gene targets, and a total of 33 putative mutations were identified based on gel analysis. All putative mutations were confirmed by Sanger sequencing to be GC-AT transition changes, the major type of change reported in species treated with EMS (Table 2). As expected, all changes were determined to be heterozygous. Analysis of the mutation spectrum revealed 36% silent, 49% mis-sense and 15% truncation alleles (see Table S3).

**Table 1:** Gene targets and primer sequences used to evaluate induced mutations in banana

Target name	Annotation	Genbank accession	Target size	Forward primer	Reverse primer
ACETRANS	N-Acetylglucosaminyltransferase	AC186756	1500	TCGCTCTGGGTTTCAGGAAAGCAGTT	TCAGAGTGTAAACCGGGGTCCCAAAT
AMTHLTR	Aminomethyltransferase	AC186747	1508	CGGCATCCAAGTTTCTCATGCCTTTTA	CAACTCGAGCAAAAAGCATCTCACGAT
DNAJ	Heatshock protein	AC186747	1473	AGGAGAAGTCAGGGACCAGAACCGAAT	TATAAACCGCCCAAATCTCACCACAGC
ELF3	Eukaryotic translation initiation factor 3	AC186746	1500	CGACTTCACAATCCCCCACATGTTAGA	GTTGTCCCCTTCAATACCGACGGATG
FTSJMT	Ftsj-like methyltransferase family protein	AC186746	1479	ATACAGCAAGGGTGATGCAGCAGACAG	ATTTGGCCTTTATTCTTGCGTCCCTTC
GHF17	Glycoside hydrolase family 17 protein	AC186755	1500	TAGGGCCAAAAGCTCCCCTGAGAAAGA	CGAGAAGGCACATAGCCGTTTCTGAGT
MALSYN	Malate synthase	AY484589	1500	CTCACCAGGGATGCCTTGCAGTTC	AGACTTCCATGATAGGCGGGATCAGG
NPH3	Nonphototropic hypocotyl 3 family protein	AC186747	1498	TCGAACCTGCTGCCAAGTTCTGTTATG	GTCCATGCTCACCTTCAAGACCTGGTT
PAAL2	Phenylalanine ammonia lyase	AP009326	1500	AGGAGGACCAAGCAAGGAGGTGCTCTT	GGTCGTCGACGTAGGTGAAGACGTG
PUF	Pumilio/Puf RNA binding	AC186756	1501	CGACGGCTTCGATGTCTACGAGTTGAT	TGGGTTGTGAGGAGAAAGTGGCTTCAC
RNDR	Ribonucleotide reductase	AY484588	1500	ATGAAGCTCCGGGACTTGCTGATTG	CAGGTTGGAGAATTCCCTGAGCAACAA

**Table 2:** Induced mutations identified in clonally propagated banana

Gene target	Nucleotide change	Mutant allele*	Effect†	PSSM‡	SIFT§
ACETRANS	M215H	C215T	Intron		
	S227B	C227T	Intron		
	C653Y	C653T	D107=		
	G1127R	G1127A	W265*		
	C1312Y	C1312T	S327F	10.8	0.08
	G1346R	G1346A	E338=		
AMTHLTR	C717Y	C717T	Intron		
	G1007R	G1007A	E267=		
DNAJ	G239R	G239A	W547*		
ELF3	G493R	G493A	S342=		
	C1040Y	C1040R	G160D		0.47
	C1092Y	C1092T	A143T	5.9	0.55
	C1107Y	C1107T	A138T	8.1	0.56
	G1119R	G1119A	R134W	6.6	0.04
	G1126R	G1126R	V131=		
	G1138R	G1138A	R127=		
	G1148R	G1148A	P124L		1.00
	C1150Y	C1150T	L123=		
FTSJMT	G284R	G284A	G485E	−2.8	1.00
	C623Y	C623T	P560S	6.3	0.27
	C986Y	C986T	Q681*		
GHF17	C1148Y	C1148T	P538S	11.5	0.04
MALSYN	C182Y	C182T	P88L	12.8	0.07
	G685R	G685A	R172Q	27	0
	G1309R	G1309A	Intron		
NPH3	G270R	G270A	A217T		
	G398R	G398A	W259*		
PAAL2	G240R	G240A	V158I	2.6	0.49
	G493R	G493A	G242D		0.09
	C696Y	C696T	Q310*		
PUF	C183Y	C183T	T714I		0.02
	G392R	G392A	G784R		0.15
RNDR	G824R	G824A	R511=		

* Owing to natural heterozygosity and triploidy, up to three alleles can be observed at any locus. Nucleotide position is based on amplicon sequence.

† Positions of changes on the amino acid sequence.

‡ Mis-sense changes are predicted to be damaging to the encoded protein if the PSSM (PARSESNP) score is >10.

§ Mis-sense changes are predicted to be damaging to the encoded protein if the SIFT score is <0.05.

### Rate of genotypic homogeneity in meristematic cells, mutation density and inheritance

Mutation densities from TILLING screens of sexually propagated species are typically calculated by counting mutations from a single progeny or pooled progeny from a mutation event, thus allowing an estimation by dividing the mutations discovered by the total bases screened (Till *et al.*, 2003). To calculate mutation densities in *in vitro* mutagenized and mitotically propagated plants, it is first necessary to evaluate at what stage in propagation the pool of meristematic cells became nonchimeric. This is required because, after this point, all sibling progeny plants within a line will be clonally related and inherit the same alleles. Including clonally related plants will result in an inflation of the population size and thereby an underestimation of mutation density when compared to estimations from seed-propagated crops. To calculate the percentage of sibling plants in the M_1_V_6_ population inheriting the same mutation, we selected 16 mutant alleles for further testing. Sibling plants were then screened individually for the presence or absence of the mutant allele. A total of 285 plants were tested and mutant alleles were recovered in ∼82% (235/285) of the plants (Table 3). In one case, the same allele (FTSJMT C623T) was identified in a subset of siblings from three different lines. While coincident mutations creating the same allele are expected to occur at a low frequency with random mutagens such as EMS, the recovery of three coincident mutations in a set of 33 discovered alleles is exceedingly unlikely. More likely, the presence of this allele in three unique lines represents human error when labelling plant flasks. Removing these potential errors from the evaluation, 222/243 (91%) of sibling plants within the population shared the same allele. This suggests that the majority of induced mutations are fixed in the treated plant meristem within 1 month after mutagenesis. We used this information to correct for the population size in estimating a mitotic mutation density (MMD) and arrive at one mutation per 57 kb. To further establish that plants in M_1_V_6_ are stably inherited, we chose three sequence-confirmed alleles and passaged plants *in vitro* to the M_1_V_9_ generation. One hundred per cent (8/8) of progeny plants carried the induced allele.

**Table 3:** Inheritance of induced mutations in sibling individuals

Gene target	Allele	Line number	Number identified	Number screened
ACETRANS	C215T	MT1_5	6	6
	C227T	MT90_83	4	4
	C653T	MT47_33	10	10
	G1127A	MT49_43	10	10
	C1312T	MT73_6	9	9
AMTHLTR	C717T	MT80_53	73	74
	G1007A	MT90_83	5	5
FTSJMT	G284A	MT57_33	10	10
	C623T	MT82_73	8	13
	C623T	MT90_23	3	11
	C623T	MT94_33	2	18
	C986T	MT82_33	9	27
MALSYN	C182T	MT99_83	2	3
	G685A	MT89_53	4	4
	G1309A	MT81_63	7	7
RNDR	G824A	MT80_53	73	74
Total			235	285

### Natural mutation rate and evaluation of natural SNPs in the mutant population

We observed no evidence of spontaneous mutations in the 10 vegetative propagation cycles before mutagenesis or the six cycles postmutagenesis. This allows for an estimation of the rate of accumulation of natural mutations in cultured banana. Using the number of generations propagated and the number of base pairs screened, we estimate the spontaneous rate to be <6 × 10^−9^_._ This is consistent with a natural mutation rate estimation of 7 × 10^−9^ calculated for *Arabidopsis thaliana* (Ossowski *et al.*, 2010). While we observed no evidence of spontaneous mutations arising in tissue culture, high heterozygosity reported for bananas suggests the accumulation of spontaneous mutations over a long evolutionary history (Till *et al.*, 2010). Therefore, natural accumulation of deleterious alleles may increase the utility of using mutagenesis in diploid and polyploid vegetatively propagated plants. To evaluate this, sequence analysis was performed on 8 selected gene targets across the regions screened by enzymatic mismatch cleavage, and polymorphisms compared to the available reference sequence using the PARSESNP and SIFT algorithms (Table 4). Of 132 catalogued polymorphisms common in all plants screened, 99/132 (75%) are silent and 25% are mis-sense changes. Approximately 8% of mis-sense changes are predicted to be deleterious to protein function. Fifty-five per cent of predicted deleterious mis-sense changes are heterozygous and 45% are homozygous compared to the reference sequence. While only a section of coding region was evaluated, heterozygous putatively deleterious alleles were found in 4/8 gene targets, suggesting a high accumulation of potentially deleterious alleles genome-wide.

**Table 4:** Natural mutations identified in triploid banana cultivar Grande Naine

Gene target	Nucleotide change*	Zygosity†	Effect‡	PARSESNP§	SIFT¶
ACETRANS	T74K	Het	S40A	10.7	0.00
	T81Y	Het	F42S	19.7	0.00
	G86K	Het	A44S	11.5	0.00
	C1076T	Hom	Y248=		
	G1295A	Hom	S321=		
AMTHLTR	A138M	Het	Intron		
	G212A	Hom	Intron		
	T220C	Hom	Intron		
	A251R	Het	Intron		
	C269M	Het	Intron		
	G277A	Hom	Intron		
	A314G	Hom	Intron		
	G347A	Hom	Intron		
	C359M	Het	Intron		
	A360G	Hom	Intron		
	G427R	Het	Intron		
	T461A	Hom	Intron		
	A522R	Het	Intron		
	A530W	Het	Intron		
	G533R	Het	Intron		
	A585W	Het	Intron		
	T589W	Het	Intron		
	G591K	Het	Intron		
	T608G	Hom	Intron		
	A615R	Het	Intron		
	A650M	Het	Intron		
	A663R	Het	Intron		
	T790W	Het	Intron		
	G822K	Het	Intron		
	C823Y	Het	Intron		
	G838R	Het	Intron		
	T850K	Het	Intron		
	G882R	Het	Intron		
	G962R	Het	S252=		
	A1256M	Het	P350=		
ELF3	A73G	Hom	I482=		
	G451A	Hom	D356=		
	A459T	Hom	S354T	−3.8	1
	G649A	Hom	V290=		
	C685T	Hom	R278=		
	G706C	Hom	S271=		
	G716A	Hom	A268V	10.5	0.09
	G778C	Hom	S247=		
	A874G	Hom	L215=		
	C964G	Hom	T185=		
	T1065G	Hom	N152H		0.24
	C1070T	Hom	R150H		0.13
	T1114C	Hom	K135=		
	C1207T	Hom	K104=		
	C1275G	Hom	V82L		0.75
	G1297A	Hom	R74=		
	C1381G	Hom	S46=		
FTSJMT	C252T	Hom	D474=		
	A323C	Hom	K498T	12.6	0.47
	A392T	Hom	Intron		
	T702A	Hom	V586E	2.3	0.96
	G763T	Hom	E606D	−5.1	0.95
	A1038G	Hom	K698R	−3.5	1
	T1177A	Hom	S744=		
	T1281G	Hom	V779G	0.7	0.58
	T1354G	Hom	G803=		
	G1369A	Hom	K808=		
MALSYN	A90R	Het	V57=		
	C119Y	Het	P67L	6.4	1
	G163R	Het	A82T	−6.1	0.67
	T180C	Hom	P87=		
	C222M	Het	V101=		
	G313R	Het	Intron		
	C331M	Het	Intron		
	C386Y	Hom	Intron		
	T640W	Het	V157E		1
	C737Y	Het	I189=		
	T872C	Hom	Intron		
	T884K	Het	Intron		
	A905G	Hom	Intron		
	A915G	Hom	Intron		
	G928A	Hom	Intron		
	A934C	Hom	Intron		
	G959S	Het	V235L	18	0.01
	T990C	Hom	I245T	18.1	0
	C1004A	Hom	L250I	−16.2	1
	G1039C	Hom	A261=		
	T1153C	Hom	D299=		
	G1239R	Het	R328H	−0.6	0.1
	C1240T	Hom	R328=		
	G1290T	Hom	Intron		
	G1320C	Hom	Intron		
	C1342T	Hom	Intron		
	G1424R	Het	K361=		
NPH3	G186R	Het	A189T	16.6	0
	A256C	Hom	Y212S	−4.9	0.86
	T576C	Hom	S319P		0.3
	C722Y	Het	S367=		
	G860R	Het	E413=		
	C1045S	Het	A475G	9.4	0.13
	A1064R	Het	R481=		
PAAL2	C75Y	Het	Intron		
	T132:5	Hom	Intron		
	A138G	Hom	Intron		
	G140:5	Hom	Intron		
	A151G	Hom	Intron		
	T167Y	Het	N133=		
	T179C	Hom	F137=		
	A480G	Hom	S238G	−10.7	1
	G503R	Het	E245=		
	G533R	Het	V255=		
	T591C	Hom	L275=		
	C602M	Het	L278=		
	A759M	Het	K331Q		0.8
	G827T	Hom	P353=		
	A870R	Het	K368E	17.4	0.04
	T941C	Hom	L391=		
	C977Y	Het	V403=		
	G1019R	Het	K417=		
	C1052Y	Het	N428=		
	T1076C	Hom	P436=		
	G1094R	Het	G442=		
	G1160A	Hom	E464=		
	T1286C	Hom	L506=		
	A1394G	Hom	R542=		
PUF	C130S	Het	A696=		
	C225Y	Het	A728V		0.06
	G279A	Hom	R746H		0.02
	C408Y	Het	T789I		0.07
	A420G	Hom	Q793R		0.02
	T436Y	Hom	C798=		
	A467M	Het	R809=		
	G497K	Het	A819S	2.4	0.16
	C556Y	Het	F838=		
	T705Y	Het	Intron		
	C719Y	Het	Intron		
	T853G	Hom	S908A		0.36

* :5, five base pairs deleted.

† Het, heterozygous, Hom, homozygous.

‡ Positions of changes on the amino acid sequence.

§ Mis-sense changes are predicted to be damaging to the encoded protein if the PARSESNP score is >10.

¶ Mis-sense changes are predicted to be damaging to the encoded protein if the SIFT score is <0.05.

## Discussion

The use of induced mutations for gene function analysis and crop improvement has been widely employed in angiosperms. Reverse genetic studies of seed-propagated plants have resulted in the production of large data sets on the effects of chemical mutagens on plant genomes. Such studies have involved screening plants after at least one meiotic event postmutagenesis. The work presented here focused on the evaluation of vegetatively propagated banana, whereby mutations were screened prior to meiosis. Chemical mutagens such as EMS have been shown to induce mutations randomly across genomes (Greene *et al.*, 2003). When mutagenizing multicellular tissues such as animal embryos, plant seed or meristems, the treated cells will accumulate different mutations and the resulting organisms will be genotypically heterogeneous or chimeric. Genetic or phenotypic measurement of such individuals is considered inappropriate because measured changes are potentially not heritable due to the fact that only a subset of cells are involved in gametogenesis. This issue has been overcome by performing at least one sexual cross prior to analysis. For plants, the single-cell spore phase ensures that progeny are genotypically homogeneous. In reverse genetic projects such as TILLING, it is often advantageous to measure the first nonchimeric generation (M_2_) to maximize the number of unique mutations recovered in the least number of samples. Evaluation of mutation segregation from a self-cross of the M_2_ generation often yields expected Mendelian segregation. However, differences in the genetically effective cell number have been observed in plants, which lead to altered segregation ratios (Greene *et al.*, 2003; Perry *et al.*, 2009).

One major difference in asexually propagated plants is the lack of passage of genetic material through the male and female haploid gametic phases. Continual mitotic propagation typically occurs in multicellular tissues. Making plants nonchimeric is therefore potentially more difficult. Chimerism in asexually propagated plants has long been observed and exploited for the generation of commercially important traits such as leaf variegation and flower colour (Marcotrigiano and Gradziel, 1997). Models for mechanisms of chimera disassociation and mosaic inheritance have been informed by observable phenotypic traits such as leaf mottling, but the underlying genetic mechanisms are often not well defined. Possible causes for such phenotypic abnormalities include transposable genetic elements, plastid mutations and mutations in nuclear genes. Phenotypic evaluations can also be influenced by somaclonal variation, a phenomenon whereby extended tissue culture can generate phenotypes, potentially influenced by epigenetic changes in cultured plants (Kaeppler *et al.*, 2000). The work presented here specifically focuses on the effect of EMS treatment on genomic DNA sequence and therefore allows for a less-biased investigation of the inheritance of genetic variants under asexual propagation. One advantage of using EMS as a mutagen is that it produces primarily GC-AT transition mutations because of the alkylation of the guanine residue and subsequent fixation of the base change during replication. For plants like *Arabidopsis thaliana* and *Triticum aestivum*, 100% transition changes have been reported. In some species such as *Drosophila melanogaster*, the recovery of fewer transition mutations may be driven by selective pressures from maintaining living populations (Cooper *et al.*, 2008). In the work presented here, screening an EMS-mutagenized banana population resulted in the recovery of 100% transition mutations. This suggests that there is limited meiotic selection against transversion mutations or small indels in previously tested TILLING populations. Another important metric in evaluating the effect of mutagenesis and the usability of mutant population is mutation density. To make an accurate estimation of mutation density in mitotically propagated plants, it is necessary to consider the genetic relationship of the siblings screened. Siblings in the M_1_V_6_ were chosen because a previous study in banana suggested that most chimeras should be dissolved before this generation, likely in the M_1_V_3_ or M_1_V_4_ (Roux *et al.*, 2001). However, after mutations are fixed and plants are genotypically homogenous, all subsequent siblings will be genetic clones and this must be corrected for when estimating mutation density. For example, if chimeras were dissolved in the M_1_V_3_ generation, it is expected that 25% of siblings in the M_1_V_6_ generation would be clonally related and share the same mutations. Upon investigation of inheritance of mutations in siblings, we observed that, in most lines, all siblings inherited the same mutations and on average approximately 90% of siblings inherit the same allele. Such a measurement reflects the genetic composition of the totipotent shoot apical meristem cells that divide to produce all above-ground tissues in the banana plant. Thus, the measurement of inheritance in the M_1_V_6_ shows that most totipotent cells were genetically homogeneous in the M_1_V_1_ 1 month after mutagenesis just prior to meristem isolation and bisection to generate the M_1_V_2_ plants.

This intriguing result was not expected based on observations that cells in the central meristematic zone (CZ) maintain their position and competency over time (Carles and Fletcher, 2003). Studies of *CLAVATA3* expression in *Arabidopsis thaliana,* a marker for the CZ, suggest that there are approximately 35 stem cells in the shoot apical meristem (Yadav *et al.*, 2009). Efforts are ongoing to accurately map and computer model the behaviour and gene expression of the shoot apical meristem (SAM). Past studies suggest that tissues are derived from at least 2–3 stem cells in CZ (Gross-Hardt and Laux, 2003). Based on our observations, we propose the following model of meristematic cell behaviour after treatment with EMS. In this model, all cells in the treated tissue accumulate transition mutations randomly throughout their nuclear genomes. Resulting cells are therefore genotypically distinct from one another, and each cell harbours a different fitness for cell division. This would result in a competitive environment where the most rapidly dividing cell eventually populates the apical meristematic region. Competitive cell divisions have been termed ‘diplontic drift’ or ‘diplontic selection’ to explain the instability of some phenotypes in vegetatively propagated plants (Balkema, 1972; Gaul, 1958; Klekowski and Kazarinovafukshansky, 1984). While previous work evoked nonmutagenized cells outcompeting cells that have naturally accumulated one or more deleterious alleles, our study allows monitoring of induced mutations, including silent mutations, accumulating in all cells without the need of observable phenotypic variation. Likely in addition to differences in cell division, the EMS treatment results in some level of cell death. This was postulated in EMS studies in *Arabidopsis thaliana* (Irish and Sussex, 1992). While we have no measure of cell death in mutagenized banana, this is not incompatible with our proposed model. A mechanism of competitive division or cell death would not be necessary if only one cell in the central zone were contributing to differentiated leaf tissue. However, if only one cell were involved, previously reported chimerism in banana would not have been observable. This therefore supports multiple cells contributing to developing primordia in *Musa acuminata*. Owing to the wide reproducibility of the effects of EMS mutagenesis in seed-propagated plants, we postulate that phenomena similar to that observed in banana may be observable in other vegetatively propagated plants. Furthermore, the same process may occur in any mutagenized meristem, including those in seed. This has practical applications when developing TILLING projects because considerable resources are required to generate a structured M_2_ population in seed-propagated plants. If rapid selection is occurring after seed mutagenesis, cells may become genotypically homogenous in the M_1_, allowing the development of DNA libraries from later leaf tissues of the first generation. Rapid mutation discovery would allow the propagation of only those M_2_ seed-harbouring desired alleles. We are currently testing this in seed-propagated crops.

We consider several mechanisms for the observed rapid genotypic homogeneity in the M_1_V_1_ generation. First, cytotoxic effects of alkylating agents may affect the competency of cells to divide (Britt, 1996). Differential competencies could be driven by variations in cytotoxicity because of cell cycle stage, activity of repair mechanisms, or by variations in EMS absorbance influenced by cellular position. Secondly, we consider genotoxic effects. While dramatic genetic changes such as chromosomal deletions and aneuploidy could be envisioned to explain variations in cellular division competencies, the large body of data from prokaryotic, eukaryotic and transgene reporter assays suggest that EMS is inducing almost exclusively point alleles (Kurowska *et al.*, 2011; Ohta *et al.*, 2000; Yoshihara *et al.*, 2006). When evaluating natural SNPs in the mutagenized banana cultivar, we observed that many gene targets harboured at least one potentially deleterious allele in the relatively small region of sequence interrogated. Obligate vegetatively propagated species have no mechanism to remove deleterious alleles and therefore will continue to accumulate them until some external pressure is applied, such as human selection. Plants may be maintained so long as at least one functional copy of essential genes is present. Such functional haploidy would be revealed in the M_1_ generation via mutagenesis where induced heterozygous point mutations could result in expressed phenotypes in otherwise recessive traits. Therefore, it is possible that genetic factors contribute to the observed rapid clearing of chimeric sectors. Interestingly, we observed 99/109 plants from 27 lines showing some phenotypic deviation from wild type in the M_1_V_10_ generation (see Table S4). Most differences were in leaf pigmentation and were variable during glasshouse growth. This could be due to the effect of induced mutations, environmental factors or somaclonal variation. However, one possible mechanism for somaclonal variation, induction of spontaneous mutation, was not observed in this population. Leaf morphology differences did not change during extended glasshouse growth. For example, a narrow leaf phenotype was observed in line MT90-83 in 26/28 (93%) of siblings assayed. This is consistent with genotypic segregation of 100% measured in this line in the M_1_V_6_ (Table 3). On average, phenotypic inheritance was observed in 91% of siblings (see Table S4), similar to genotypic measurements, supporting the model of rapid fixation of meristematic cells after mutagenesis. A larger-scale genomic analysis is required to determine whether mutations affecting gene functions are driving genotypic homogeneity in mutagenized banana cultures. This will be aided by the recent release of the banana genome sequence (D’Hont *et al.*, 2012).

Analysis of inheritance of induced mutations in siblings allowed a correction for population size and an estimation of mutation density analogous to those reported in sexually propagated plants and animals. We calculate a density of 1 mutation per 57 kb. With a genome size of approximately 520 Mb (D’Hont *et al.*, 2012), we estimate that on average each plant carries approximately 10 000 induced alleles. While DNA sequence-based mutation densities have not previously been reported for triploids, the density is higher than that reported for diploids but lower than tetraploid and hexaploid wheat that was screened using the same mutation discovery protocol. This controls for variations in ascertainment biases in different mutation discovery methods (Jankowicz-Cieslak *et al.*, 2011). The calculated estimate is expected as increasing copy number provides redundancy that protects against mutagens, thus allowing higher mutation densities to be achieved with increasing ploidy (Slade *et al.*, 2005; Stadler, 1929; Uauy *et al.*, 2009). That the expected density was recovered in mitotically propagated tissues suggests that treatment of isolated meristems in liquid EMS was near optimal. The expected triploid mutation density is based on sexually propagated species, suggesting that most of the mutations induced in vegetatively propagated tissues are meiotically heritable. This has practical implications for applications in facultative vegetatively propagated plants. Finally, we showed that induced alleles remain stable in culture conditions through ten cycles of propagation.

The work presented suggests strategies that may be applicable for a range of vegetatively propagated plant species, including those of commercial importance (potato, hops, grapevine, citrus) and those of importance for food security in developing countries (cassava, sweet potato, banana). Further experiments are ongoing to test our models in other asexually propagated species. The utility of induced mutations and reverse genetics may potentially be of greatest applicability in facultative vegetatively propagated crops where meiosis is possible, but either inefficient or seldom used. Here, tissue culture mutagenesis could be employed to produce nonchimeric M_1_ populations rapidly for genotypic mutation screening at a core facility. Only plants containing desired mutants need to be selected and delivered for self-crossing and field evaluation. Thus, the effective population size compared to forward genetic mutation breeding approaches could be reduced by several orders of magnitude, and bottlenecks in sexual propagation, such as low fecundity, can be overcome by clonal propagation. Because standardized *in vitro* propagation protocols can be applied to many species, and vegetative material can easily be shipped as micropropagules, a single facility could service a wide geographical range. The utility of point mutations for trait development in obligate vegetatively propagated plants and the prevalence of functional haploidy remain to be further investigated. Alternative mutagenesis approaches, such as inducing aneuploidy, may provide a means to generate more observable phenotypes in plants where meiotic propagation is not possible (Henry *et al.*, 2010). Tissue culture has been established for many plant species, and the methods described here, where a mutant population can be rapidly produced, may also prove highly advantageous for seed-propagated species, as these approaches uncouple the dependency on field propagation and shorten the time until desired alleles are recovered.

## Experimental procedures

### Plant material

*In vitro* plantlets of banana (*Musa acuminata*) ecotype Grande Naine (Cavendish AAA, ITC accession 0180) were obtained from the Bioversity’s International Transit Centre (http://www.crop-diversity.org/banana/#AvailableITCAccessions, accessed 8 March 2012). Plants were maintained *in vitro* through aseptic shoot tip cultures in liquid S27 media supplemented with 20 μm 6-benzylaminopurine, 1 mg/L thiamine, 40 mg/L cystein HCl and 40 g/L sucrose, pH 5.8. The liquid cultures were maintained under constant horizontal rotation at 60/min with a continuous light (65 μmol/m^2^/s; Cool White fluorescent tubes, Philips TLP 36/86, Philips, Amsterdam, the Netherlands) at 22° ± 2°. Shoot multiplication was induced by cutting a single isolated shoot tip longitudinally through the apex using a scalpel blade (Cronauer and Krikorian, 1984). The resulting two shoot halves were placed in freshly prepared liquid media for 3–4 weeks to allow shoot regeneration. At this point, outer leaves produced from the dividing shoot tip and the shoot bases were trimmed away. Shoot isolation and division was then carried out. This process was repeated for a total of 10 generative cycles until the number of clonal propagules reached 4400.

### Optimization of chemical mutagenesis

To determine a dosage range for the treatment of banana with ethyl methanesulphonate (EMS), shoot tips were incubated for either 2 or 4 h in 0.25%, 0.5%, 1% and 1.5% concentrations of mutagen. For each treatment combination, 25 *in vitro* cuttings were soaked in EMS solution of the selected percentage in sterilized water plus 2% dimethyl sulphoxide (v/v, DMSO). Control material was treated in parallel under similar conditions minus addition of EMS. After the treatment, shoot tips were rinsed a minimum of five times in distilled water and subsequently placed into culture media. After 24 h, mutagenized tissue was transferred into fresh media and incubated for an additional 30 days as described above. After this period, the fresh weight of mutagenized and nonmutagenized tissue was recorded and survivability measured. This was used as a qualitative measure of response to mutagenic treatment. To evaluate the effect of mutagenesis on sample weight, analysis of variance (ANOVA) was conducted using Statistica 9.0 software (StatSoft®, Tulsa, OK). Significant differences were observed between treated and control samples (*P* = 0.05). This was used to select parameters for bulk mutagenesis.

### Bulk mutagenesis

To balance mutation density and survivability, a series of treatments was applied. A total of 4000 shoot tips were treated with EMS in 1000 sample batches for 3 h (1% EMS), 6 h (0.5% EMS), 24 h (0.125% EMS) and 48 h (0.06% EMS). After mutagenic treatment, groups of 10 isolated shoot tips were placed in individual culture flasks. Each shoot tip cutting was defined as the progenitor material for a subsequent set of related sibling progeny (referred to as a line). Cultures were transferred weekly into fresh liquid media to reduce possible accumulation of phenolic compounds because of the stress of mutagenesis. Thirty days after the treatment, survival rates of the mutagenized population were recorded. At this point, each progenitor *in vitro* plantlet was transferred into a separate flask with fresh media and assigned a unique number that represents the line number for siblings that will be produced from this individual. The progenitor material was labelled M_1_V_1_. V_1_ signifies the first vegetative generation after mutagenic treatment. Increasing numbers following V represent successive vegetative generations, and increasing numbers after M indicate meiotic propagation. This allows tracking of generations in both facultative and obligate vegetatively propagated species. To remove potential chimeric sectors caused by the random mutagenesis of different cells in the shoot tip, repeated propagation was carried out through the successive division of apical shoot tip meristems as described above. At each division, material was transferred into fresh culture media and allowed to recover for 4–5 weeks. This process was repeated five times to make a population of M_1_V_6_ individuals (Figure 1). From this, a total of 1000 plants were randomly selected for DNA extraction and mutation screening. These plants were transferred to solid media containing culture media plus 2.2% Gelrite and maintained at 22° ± 2° under stationary incubation and 12 h light cycle for the duration of the study.

### Genomic DNA isolation

Approximately 50 mg of fresh leaf tissue was harvested from the randomly selected M_1_V_6_ plants, and genomic DNA was extracted using the DNeasy 96 Plant Kit (Qiagen, Valencia, CA). Sample quality and quantity was evaluated using standard agarose gel assays and normalized to a concentration of 3 ng/μL. Seven hundred and sixty-eight DNA samples were arrayed and pooled eightfold using a two-dimensional pooling strategy (Till *et al.*, 2006). Samples were diluted to 0.1 ng/μL in Tris–EDTA (TE) buffer prior to PCR amplification.

### Primer design, TILLING and evaluation of induced and natural mutations

Primers were designed for 23 genic target regions from nine publically available BAC clones using the CODDLe and Primer 3 software with default parameters for TILLING assays (Till *et al.*, 2006). Melting temperatures ranged between 67° and 73° (Table 1). PCR amplification, enzymatic mismatch cleavage using a crude celery juice extract, mutation detection on a LI-COR DNA analyzer, data analysis using GelBuddy software and sequence validation of identified mutations was performed as previously described (Till *et al.*, 2006). The effect of induced and natural mutations on protein sequence was evaluated using the PARSESNP programme that incorporates the SIFT algorithm (Ng and Henikoff, 2006; Taylor and Greene, 2003). Nucleotide positions for alleles were derived from annotated regions of reference sequences listed in Table 1. Sequences of induced and natural alleles are deposited in GenBank (http://www.ncbi.nlm.nih.gov/) with accession numbers JX123279 through JX123314.

### Estimation of mutation density

Population mutation density was calculated by dividing the total number of sequence-validated mutations by the total number of bases screened (sum of amplicon sizes multiplied by total number of samples screened). This was corrected for the terminal 100 base pairs on each fragment end where mutation discovery is hindered (Greene *et al.*, 2003). Because sibling plants derived from a single meristem may be clonally related and harbouring the same mutations, mutation density was adjusted by estimating the number of genotypically unique individuals in the screened population. This was carried out by screening multiple sibling plants of the same lineage to evaluate the segregation of induced mutations (Table 3). The resulting population size of unique individuals was used in the calculation of mutation density. A further correction factor for mutation density was applied because mutation screening was performed simultaneously on all three genomes rather than screening only one homeologue in a polyploid species as previously described (Slade *et al.*, 2005). Accurate recovery of single nucleotide variations by simultaneous screening of homeologous sequences was previously shown in studies evaluating natural nucleotide variation in Musa (Till *et al.*, 2010). The calculation used for mutation density is as follows:


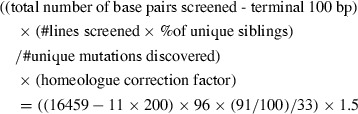


To differentiate between mutation densities reported in TILLING literature from meiotically propagated plants and animals, we refer to this adjusted mutation density as MMD (mitotic mutation density).

### Heritability of induced mutations

Plants harbouring three confirmed mutant alleles (MALSYN G1309A, FTSJMT C623T and FTSJMT C986T) were selected to evaluate the heritability of induced mutations in culture conditions. *In vitro* plantlets were propagated and maintained in solid media for a period of 12 months up to the M_1_V_9_ generation. Presence or absence of the induced allele was evaluated using Sanger sequencing.

### Phenotypic evaluation of mutagenized plants

One hundred and nine randomly selected plantlets representing 27 mutant lines were propagated to the M_1_V_10_ generation as described above and transferred to the glasshouse in pots containing Torboton I soil (Gartenhilfe Ges.m.b.H, Linz, Austria). Plants were acclimatized to standardized glasshouse conditions with average temperature of 25° and 40%–50% air humidity for 24 months before measurements were recorded. Phenotypical observations were taken following standardized banana descriptors (http://cropgenebank.sgrp.cgiar.org/images/file/learning_space/descriptors_banana.pdf, accessed 8 August 2012).

## Supporting information

**Table S1** Plant survival after treatment with EMS.

**Table S2** Mutagenesis treatment and line numbers of banana samples included in this study.

**Table S3** Expected and observed mutations for tested amplicons.

**Table S4** Frequencies of observed phenotypesafter 24 months greenhouse growth.
