# Supplementary material for: Induction, rapid fixation and retention of mutations in vegetatively propagated banana
Source: Plant Biotechnol J. 2012 Dec;10(9):1056–66. doi: 10.1111/j.1467-7652.2012.00733.x (PMC3533788; doi:10.1111/j.1467-7652.2012.00733.x)
Supplement: Supplementary file 1 [file pbi0010-1056-SD1.doc]

| **Table S1** Plant survival after treatment with EMS | | | |  |
| --- | --- | --- | --- | --- |
|  |  |  |  |  |
| **EMS (%)** | **Time (hours)** | **Number treated** | **Number survived^a^** | **Survival (%)** |
| 0 (control) | 0 | 100 | 100 | 100 |
| 1 | 3 | 990 | 844 | 85 |
| 0.5 | 6 | 997 | 802 | 80 |
| 0.125 | 24 | 991 | 513 | 52 |
| 0.063 | 48 | 976 | 845 | 87 |
|  |  |  |  |  |
|  |  |  |  |  |
| ^a^Survival measured one month after treatment | | |  |  |
