# Supplementary material for: Induction, rapid fixation and retention of mutations in vegetatively propagated banana
Source: Plant Biotechnol J. 2012 Dec;10(9):1056–66. doi: 10.1111/j.1467-7652.2012.00733.x (PMC3533788; doi:10.1111/j.1467-7652.2012.00733.x)
Supplement: Supplementary file 2 [file pbi0010-1056-SD2.doc]

**Table S2** Mutagenesis treatment and line numbers of banana samples included in this study

| **DNA Sample #** | **EMS treatment** | **Line (Pedigree designation)** |
| --- | --- | --- |
| bt_0754 | 3h_1% | MT1_13 |
| bt_0538 | 48h_0.06% | MT1_24 |
| bt_0539 | 48h_0.06% | MT1_24 |
| bt_0540 | 48h_0.06% | MT1_24 |
| bt_0193 | 48h_0.06% | MT1_24 |
| bt_0194 | 48h_0.06% | MT1_24 |
| bt_0195 | 48h_0.06% | MT1_24 |
| bt_0196 | 48h_0.06% | MT1_24 |
| bt_0954 | 48h_0.06% | MT1_24 |
| bt_0955 | 48h_0.06% | MT1_24 |
| bt_0957 | 48h_0.06% | MT1_24 |
| bt_0134 | 3h_1% | MT1_53 |
| bt_0135 | 3h_1% | MT1_53 |
| bt_0136 | 3h_1% | MT1_53 |
| bt_0137 | 3h_1% | MT1_53 |
| bt_0138 | 3h_1% | MT1_53 |
| bt_0139 | 3h_1% | MT1_53 |
| bt_0197 | 3h_1% | MT1_53 |
| bt_0199 | 3h_1% | MT1_53 |
| bt_0692 | 3h_1% | MT1_53 |
| bt_0097 | 48h_0.06% | MT12_84 |
| bt_0098 | 48h_0.06% | MT12_84 |
| bt_0099 | 48h_0.06% | MT12_84 |
| bt_0100 | 48h_0.06% | MT12_84 |
| bt_0101 | 48h_0.06% | MT12_84 |
| bt_0189 | 48h_0.06% | MT12_84 |
| bt_0190 | 48h_0.06% | MT12_84 |
| bt_0191 | 48h_0.06% | MT12_84 |
| bt_0192 | 48h_0.06% | MT12_84 |
| bt_0288 | 24h_0.125% | MT12_92 |
| bt_0856 | 24h_0.125% | MT14_182 |
| bt_0541 | 24h_0.125% | MT15_102 |
| bt_0543 | 24h_0.125% | MT15_102 |
| bt_0767 | 24h_0.125% | MT15_102 |
| bt_0536 | 48h_0.06% | MT16_14 |
| bt_0537 | 48h_0.06% | MT16_14 |
| bt_0784 | 6h_0.5% | MT18_16 |
| bt_0204 | 48h_0.06% | MT2_54 |
| bt_0205 | 48h_0.06% | MT2_54 |
| bt_0206 | 48h_0.06% | MT2_54 |
| bt_0142 | 3h_1% | MT2_53 |
| bt_0143 | 3h_1% | MT2_53 |
| bt_0144 | 3h_1% | MT2_53 |
| bt_0533 | 3h_1% | MT2_53 |
| bt_0534 | 3h_1% | MT2_53 |
| bt_0535 | 3h_1% | MT2_53 |
| bt_0697 | 6h_0.5% | MT22_26 |
| bt_0698 | 6h_0.5% | MT22_26 |
| bt_0699 | 6h_0.5% | MT22_26 |
| bt_0209 | 48h_0.06% | MT26_44 |
| bt_0210 | 48h_0.06% | MT26_44 |
| bt_0211 | 48h_0.06% | MT26_44 |
| bt_0212 | 48h_0.06% | MT26_44 |
| bt_0411 | 3h_1% | MT3_33 |
| bt_0412 | 3h_1% | MT3_33 |
| bt_0413 | 3h_1% | MT3_33 |
| bt_0414 | 3h_1% | MT3_33 |
| bt_0415 | 3h_1% | MT3_33 |
| bt_0416 | 3h_1% | MT3_33 |
| bt_0418 | 3h_1% | MT3_33 |
| bt_0419 | 3h_1% | MT3_33 |
| bt_0420 | 3h_1% | MT3_33 |
| bt_0421 | 3h_1% | MT3_33 |
| bt_0422 | 3h_1% | MT3_33 |
| bt_0423 | 3h_1% | MT3_33 |
| bt_0424 | 3h_1% | MT3_33 |
| bt_0426 | 3h_1% | MT3_33 |
| bt_0427 | 3h_1% | MT3_33 |
| bt_0428 | 3h_1% | MT3_33 |
| bt_0429 | 3h_1% | MT3_33 |
| bt_0843 | 3h_1% | MT3_33 |
| bt_0844 | 3h_1% | MT3_33 |
| bt_0845 | 3h_1% | MT3_33 |
| bt_0528 | 48h_0.06% | MT3_44 |
| bt_0529 | 48h_0.06% | MT3_44 |
| bt_0530 | 48h_0.06% | MT3_44 |
| bt_0531 | 48h_0.06% | MT3_44 |
| bt_0532 | 48h_0.06% | MT3_44 |
| bt_0076 | 3h_1% | MT33_63 |
| bt_0077 | 3h_1% | MT33_63 |
| bt_0078 | 3h_1% | MT33_63 |
| bt_0079 | 3h_1% | MT33_63 |
| bt_0080 | 3h_1% | MT33_63 |
| bt_0081 | 3h_1% | MT33_63 |
| bt_0082 | 3h_1% | MT33_63 |
| bt_0083 | 3h_1% | MT33_63 |
| bt_0166 | 3h_1% | MT33_63 |
| bt_0167 | 3h_1% | MT33_63 |
| bt_0168 | 3h_1% | MT33_63 |
| bt_0169 | 3h_1% | MT33_63 |
| bt_0170 | 3h_1% | MT33_63 |
| bt_0171 | 3h_1% | MT33_63 |
| bt_0172 | 3h_1% | MT33_63 |
| bt_0173 | 3h_1% | MT33_63 |
| bt_0174 | 3h_1% | MT33_63 |
| bt_0175 | 3h_1% | MT33_63 |
| bt_0176 | 3h_1% | MT33_63 |
| bt_0376 | 3h_1% | MT33_63 |
| bt_0377 | 3h_1% | MT33_63 |
| bt_0378 | 3h_1% | MT33_63 |
| bt_0379 | 3h_1% | MT33_63 |
| bt_0380 | 3h_1% | MT33_63 |
| bt_0786 | 3h_1% | MT33_63 |
| bt_0788 | 3h_1% | MT33_63 |
| bt_0789 | 3h_1% | MT33_63 |
| bt_0772 | 24h_0.125% | MT35_22 |
| bt_0773 | 24h_0.125% | MT35_22 |
| bt_0775 | 24h_0.125% | MT35_22 |
| bt_0574 | 6h_0.5% | MT38_16 |
| bt_0575 | 6h_0.5% | MT38_16 |
| bt_0102 | 48h_0.06% | MT38_104 |
| bt_0103 | 48h_0.06% | MT38_104 |
| bt_0104 | 48h_0.06% | MT38_104 |
| bt_0105 | 48h_0.06% | MT38_104 |
| bt_0106 | 48h_0.06% | MT38_104 |
| bt_0685 | 24h_0.125% | MT4_52 |
| bt_0686 | 24h_0.125% | MT4_52 |
| bt_0650 | 24h_0.125% | MT40_32 |
| bt_0651 | 24h_0.125% | MT40_32 |
| bt_0605 | 3h_1% | MT41_43 |
| bt_0607 | 3h_1% | MT41_43 |
| bt_0799 | 3h_1% | MT41_63 |
| bt_0800 | 3h_1% | MT41_63 |
| bt_0585 | 3h_1% | MT41_73 |
| bt_0586 | 3h_1% | MT41_73 |
| bt_0587 | 3h_1% | MT41_73 |
| bt_0588 | 3h_1% | MT41_73 |
| bt_0589 | 3h_1% | MT41_73 |
| bt_0591 | 3h_1% | MT41_73 |
| bt_0779 | 24h_0.125% | MT43_12 |
| bt_0781 | 24h_0.125% | MT43_12 |
| bt_0732 | 48h_0.06% | MT43_54 |
| bt_0733 | 48h_0.06% | MT43_54 |
| bt_0734 | 48h_0.06% | MT43_54 |
| bt_0735 | 48h_0.06% | MT43_54 |
| bt_0737 | 48h_0.06% | MT43_54 |
| bt_0738 | 48h_0.06% | MT43_54 |
| bt_0074 | 3h_1% | MT47_33 |
| bt_0075 | 3h_1% | MT47_33 |
| bt_0145 | 3h_1% | MT47_33 |
| bt_0146 | 3h_1% | MT47_33 |
| bt_0147 | 3h_1% | MT47_33 |
| bt_0148 | 3h_1% | MT47_33 |
| bt_0149 | 3h_1% | MT47_33 |
| bt_0150 | 3h_1% | MT47_33 |
| bt_0151 | 3h_1% | MT47_33 |
| bt_0152 | 3h_1% | MT47_33 |
| bt_0153 | 3h_1% | MT47_33 |
| bt_0154 | 3h_1% | MT47_33 |
| bt_0155 | 3h_1% | MT47_33 |
| bt_0156 | 3h_1% | MT47_33 |
| bt_0157 | 3h_1% | MT47_33 |
| bt_0158 | 3h_1% | MT47_33 |
| bt_0159 | 3h_1% | MT47_33 |
| bt_0160 | 3h_1% | MT47_33 |
| bt_0161 | 3h_1% | MT47_33 |
| bt_0162 | 3h_1% | MT47_33 |
| bt_0435 | 3h_1% | MT49_43 |
| bt_0436 | 3h_1% | MT49_43 |
| bt_0437 | 3h_1% | MT49_43 |
| bt_0438 | 3h_1% | MT49_43 |
| bt_0439 | 3h_1% | MT49_43 |
| bt_0440 | 3h_1% | MT49_43 |
| bt_0441 | 3h_1% | MT49_43 |
| bt_0442 | 3h_1% | MT49_43 |
| bt_0443 | 3h_1% | MT49_43 |
| bt_0445 | 3h_1% | MT49_43 |
| bt_0622 | 24h_0.125% | MT50_92 |
| bt_0623 | 24h_0.125% | MT50_92 |
| bt_0624 | 24h_0.125% | MT50_92 |
| bt_0625 | 24h_0.125% | MT50_92 |
| bt_0626 | 24h_0.125% | MT50_92 |
| bt_0804 | 24h_0.125% | MT50_92 |
| bt_0805 | 24h_0.125% | MT50_92 |
| bt_0807 | 24h_0.125% | MT50_92 |
| bt_0084 | 48h_0.06% | MT52_34 |
| bt_0085 | 48h_0.06% | MT52_34 |
| bt_0086 | 48h_0.06% | MT52_34 |
| bt_0087 | 48h_0.06% | MT52_34 |
| bt_0088 | 48h_0.06% | MT52_34 |
| bt_0089 | 48h_0.06% | MT52_34 |
| bt_0090 | 48h_0.06% | MT52_34 |
| bt_0091 | 48h_0.06% | MT52_34 |
| bt_0092 | 48h_0.06% | MT52_34 |
| bt_0093 | 48h_0.06% | MT52_34 |
| bt_0094 | 48h_0.06% | MT52_34 |
| bt_0398 | 48h_0.06% | MT54_74 |
| bt_0400 | 48h_0.06% | MT54_74 |
| bt_0391 | 3h_1% | MT55_73 |
| bt_0392 | 3h_1% | MT55_73 |
| bt_0394 | 3h_1% | MT55_73 |
| bt_0395 | 3h_1% | MT55_73 |
| bt_0396 | 3h_1% | MT55_73 |
| bt_0809 | 24h_0.125% | MT56_12 |
| bt_0742 | 6h_0.5% | MT57_26 |
| bt_0276 | 3h_1% | MT57_33 |
| bt_0277 | 3h_1% | MT57_33 |
| bt_0278 | 3h_1% | MT57_33 |
| bt_0279 | 3h_1% | MT57_33 |
| bt_0280 | 3h_1% | MT57_33 |
| bt_0828 | 3h_1% | MT57_33 |
| bt_0829 | 3h_1% | MT57_33 |
| bt_0950 | 3h_1% | MT57_33 |
| bt_0951 | 3h_1% | MT57_33 |
| bt_0952 | 3h_1% | MT57_33 |
| bt_0953 | 3h_1% | MT57_33 |
| bt_0753 | 3h_1% | MT57_43 |
| bt_0743 | 3h_1% | MT59_63 |
| bt_0744 | 3h_1% | MT59_63 |
| bt_0745 | 3h_1% | MT59_63 |
| bt_0746 | 3h_1% | MT59_63 |
| bt_0747 | 3h_1% | MT59_63 |
| bt_0544 | 48h_0.06% | MT6_24 |
| bt_0545 | 48h_0.06% | MT6_24 |
| bt_0546 | 48h_0.06% | MT6_24 |
| bt_0140 | 3h_1% | MT62_53 |
| bt_0141 | 3h_1% | MT62_53 |
| bt_0561 | 3h_1% | MT62_53 |
| bt_0562 | 3h_1% | MT62_53 |
| bt_0563 | 3h_1% | MT62_53 |
| bt_0681 | 3h_1% | MT62_53 |
| bt_0682 | 3h_1% | MT62_53 |
| bt_0684 | 3h_1% | MT62_53 |
| bt_0765 | 3h_1% | MT62_53 |
| bt_0861 | 3h_1% | MT62_53 |
| bt_0922 | 3h_1% | MT62_53 |
| bt_0923 | 3h_1% | MT62_53 |
| bt_0924 | 3h_1% | MT62_53 |
| bt_0928 | 3h_1% | MT62_53 |
| bt_0253 | 3h_1% | MT62_73 |
| bt_0254 | 3h_1% | MT62_73 |
| bt_0255 | 3h_1% | MT62_73 |
| bt_0518 | 3h_1% | MT62_73 |
| bt_0519 | 3h_1% | MT62_73 |
| bt_0520 | 3h_1% | MT62_73 |
| bt_0521 | 3h_1% | MT62_73 |
| bt_0522 | 3h_1% | MT62_73 |
| bt_0523 | 3h_1% | MT62_73 |
| bt_0524 | 3h_1% | MT62_73 |
| bt_0525 | 3h_1% | MT62_73 |
| bt_0526 | 3h_1% | MT62_73 |
| bt_0527 | 3h_1% | MT62_73 |
| bt_0550 | 3h_1% | MT62_73 |
| bt_0551 | 3h_1% | MT62_73 |
| bt_0552 | 3h_1% | MT62_73 |
| bt_0553 | 3h_1% | MT62_73 |
| bt_0554 | 3h_1% | MT62_73 |
| bt_0555 | 3h_1% | MT62_73 |
| bt_0556 | 3h_1% | MT62_73 |
| bt_0557 | 3h_1% | MT62_73 |
| bt_0674 | 3h_1% | MT62_73 |
| bt_0700 | 3h_1% | MT62_73 |
| bt_0701 | 3h_1% | MT62_73 |
| bt_0945 | 3h_1% | MT62_73 |
| bt_0946 | 3h_1% | MT62_73 |
| bt_0948 | 3h_1% | MT62_73 |
| bt_0690 | 3h_1% | MT65_23 |
| bt_0402 | 48h_0.06% | MT66_14 |
| bt_0403 | 48h_0.06% | MT66_14 |
| bt_0404 | 48h_0.06% | MT66_14 |
| bt_0061 | 48h_0.06% | MT66_74 |
| bt_0063 | 48h_0.06% | MT66_74 |
| bt_0066 | 48h_0.06% | MT66_74 |
| bt_0095 | 48h_0.06% | MT69_34 |
| bt_0096 | 48h_0.06% | MT69_34 |
| bt_0824 | 6h_0.5% | MT70_36 |
| bt_0825 | 6h_0.5% | MT70_36 |
| bt_0827 | 6h_0.5% | MT70_36 |
| bt_0615 | 6h_0.5% | MT70_46 |
| bt_0616 | 6h_0.5% | MT70_46 |
| bt_0617 | 6h_0.5% | MT70_46 |
| bt_0618 | 6h_0.5% | MT70_46 |
| bt_0619 | 6h_0.5% | MT70_46 |
| bt_0620 | 6h_0.5% | MT70_46 |
| bt_0621 | 6h_0.5% | MT70_46 |
| bt_0849 | 48h_0.06% | MT70_94 |
| bt_0850 | 48h_0.06% | MT70_94 |
| bt_0759 | 6h_0.5% | MT71_26 |
| bt_0760 | 6h_0.5% | MT71_26 |
| bt_0796 | 6h_0.5% | MT73_16 |
| bt_0709 | 24h_0.125% | MT73_42 |
| bt_0710 | 24h_0.125% | MT73_42 |
| bt_0711 | 24h_0.125% | MT73_42 |
| bt_0712 | 24h_0.125% | MT73_42 |
| bt_0713 | 24h_0.125% | MT73_42 |
| bt_0714 | 24h_0.125% | MT73_42 |
| bt_0715 | 24h_0.125% | MT73_42 |
| bt_0705 | 6h_0.5% | MT73_46 |
| bt_0706 | 6h_0.5% | MT73_46 |
| bt_0707 | 6h_0.5% | MT73_46 |
| bt_0708 | 6h_0.5% | MT73_46 |
| bt_0184 | 24h_0.125% | MT73_62 |
| bt_0185 | 24h_0.125% | MT73_62 |
| bt_0186 | 24h_0.125% | MT73_62 |
| bt_0187 | 24h_0.125% | MT73_62 |
| bt_0188 | 24h_0.125% | MT73_62 |
| bt_0256 | 24h_0.125% | MT73_62 |
| bt_0260 | 24h_0.125% | MT73_62 |
| bt_0261 | 24h_0.125% | MT73_62 |
| bt_0263 | 24h_0.125% | MT73_62 |
| bt_0264 | 24h_0.125% | MT73_62 |
| bt_0268 | 24h_0.125% | MT73_62 |
| bt_0270 | 24h_0.125% | MT73_62 |
| bt_0271 | 24h_0.125% | MT73_62 |
| bt_0273 | 24h_0.125% | MT73_62 |
| bt_0274 | 24h_0.125% | MT73_62 |
| bt_0275 | 24h_0.125% | MT73_62 |
| bt_0935 | 24h_0.125% | MT73_62 |
| bt_0939 | 24h_0.125% | MT73_62 |
| bt_0943 | 24h_0.125% | MT73_62 |
| bt_0944 | 24h_0.125% | MT73_62 |
| bt_0071 | 48h_0.06% | MT74_64 |
| bt_0072 | 48h_0.06% | MT74_64 |
| bt_0073 | 48h_0.06% | MT74_64 |
| bt_0067 | 6h_0.5% | MT78_66 |
| bt_0068 | 6h_0.5% | MT78_66 |
| bt_0069 | 6h_0.5% | MT78_66 |
| bt_0070 | 6h_0.5% | MT78_66 |
| bt_0653 | 48h_0.06% | MT79_14 |
| bt_0654 | 3h_1% | MT79_43 |
| bt_0655 | 3h_1% | MT79_43 |
| bt_0657 | 3h_1% | MT79_43 |
| bt_0658 | 3h_1% | MT79_43 |
| bt_0666 | 48h_0.06% | MT8_14 |
| bt_0667 | 48h_0.06% | MT8_14 |
| bt_0668 | 48h_0.06% | MT8_14 |
| bt_0128 | 3h_1% | MT80_53 |
| bt_0129 | 3h_1% | MT80_53 |
| bt_0130 | 3h_1% | MT80_53 |
| bt_0132 | 3h_1% | MT80_53 |
| bt_0133 | 3h_1% | MT80_53 |
| bt_0207 | 3h_1% | MT80_53 |
| bt_0208 | 3h_1% | MT80_53 |
| bt_0359 | 3h_1% | MT80_53 |
| bt_0360 | 3h_1% | MT80_53 |
| bt_0361 | 3h_1% | MT80_53 |
| bt_0362 | 3h_1% | MT80_53 |
| bt_0366 | 3h_1% | MT80_53 |
| bt_0368 | 3h_1% | MT80_53 |
| bt_0369 | 3h_1% | MT80_53 |
| bt_0370 | 3h_1% | MT80_53 |
| bt_0371 | 3h_1% | MT80_53 |
| bt_0372 | 3h_1% | MT80_53 |
| bt_0373 | 3h_1% | MT80_53 |
| bt_0375 | 3h_1% | MT80_53 |
| bt_0873 | 3h_1% | MT80_53 |
| bt_0874 | 3h_1% | MT80_53 |
| bt_0880 | 3h_1% | MT80_53 |
| bt_0881 | 3h_1% | MT80_53 |
| bt_0884 | 3h_1% | MT80_53 |
| bt_0885 | 3h_1% | MT80_53 |
| bt_0889 | 3h_1% | MT80_53 |
| bt_0892 | 3h_1% | MT80_53 |
| bt_0895 | 3h_1% | MT80_53 |
| bt_0896 | 3h_1% | MT80_53 |
| bt_0897 | 3h_1% | MT80_53 |
| bt_0898 | 3h_1% | MT80_53 |
| bt_0900 | 3h_1% | MT80_53 |
| bt_0902 | 3h_1% | MT80_53 |
| bt_0903 | 3h_1% | MT80_53 |
| bt_0904 | 3h_1% | MT80_53 |
| bt_0906 | 3h_1% | MT80_53 |
| bt_0907 | 3h_1% | MT80_53 |
| bt_0909 | 3h_1% | MT80_53 |
| bt_0913 | 3h_1% | MT80_53 |
| bt_0915 | 3h_1% | MT80_53 |
| bt_0918 | 3h_1% | MT80_53 |
| bt_0919 | 3h_1% | MT80_53 |
| bt_0920 | 3h_1% | MT80_53 |
| bt_0003 | 3h_1% | MT80_53 |
| bt_0004 | 3h_1% | MT80_53 |
| bt_0005 | 3h_1% | MT80_53 |
| bt_0006 | 3h_1% | MT80_53 |
| bt_0007 | 3h_1% | MT80_53 |
| bt_0008 | 3h_1% | MT80_53 |
| bt_0009 | 3h_1% | MT80_53 |
| bt_0010 | 3h_1% | MT80_53 |
| bt_0011 | 3h_1% | MT80_53 |
| bt_0012 | 3h_1% | MT80_53 |
| bt_0013 | 3h_1% | MT80_53 |
| bt_0014 | 3h_1% | MT80_53 |
| bt_0015 | 3h_1% | MT80_53 |
| bt_0016 | 3h_1% | MT80_53 |
| bt_0017 | 3h_1% | MT80_53 |
| bt_0018 | 3h_1% | MT80_53 |
| bt_0019 | 3h_1% | MT80_53 |
| bt_0020 | 3h_1% | MT80_53 |
| bt_0021 | 3h_1% | MT80_53 |
| bt_0022 | 3h_1% | MT80_53 |
| bt_0023 | 3h_1% | MT80_53 |
| bt_0024 | 3h_1% | MT80_53 |
| bt_0025 | 3h_1% | MT80_53 |
| bt_0026 | 3h_1% | MT80_53 |
| bt_0027 | 3h_1% | MT80_53 |
| bt_0028 | 3h_1% | MT80_53 |
| bt_0029 | 3h_1% | MT80_53 |
| bt_0030 | 3h_1% | MT80_53 |
| bt_0031 | 3h_1% | MT80_53 |
| bt_0032 | 3h_1% | MT80_53 |
| bt_0033 | 3h_1% | MT80_53 |
| bt_0723 | 3h_1% | MT80_73 |
| bt_0724 | 3h_1% | MT80_73 |
| bt_0725 | 3h_1% | MT80_73 |
| bt_0726 | 3h_1% | MT80_73 |
| bt_0727 | 3h_1% | MT80_73 |
| bt_0241 | 3h_1% | MT81_103 |
| bt_0242 | 3h_1% | MT81_103 |
| bt_0243 | 3h_1% | MT81_103 |
| bt_0244 | 3h_1% | MT81_103 |
| bt_0245 | 3h_1% | MT81_103 |
| bt_0246 | 3h_1% | MT81_103 |
| bt_0247 | 3h_1% | MT81_103 |
| bt_0248 | 3h_1% | MT81_103 |
| bt_0249 | 3h_1% | MT81_103 |
| bt_0250 | 3h_1% | MT81_103 |
| bt_0251 | 3h_1% | MT81_103 |
| bt_0252 | 3h_1% | MT81_103 |
| bt_0381 | 3h_1% | MT81_103 |
| bt_0382 | 3h_1% | MT81_103 |
| bt_0383 | 3h_1% | MT81_103 |
| bt_0384 | 3h_1% | MT81_103 |
| bt_0820 | 3h_1% | MT81_103 |
| bt_0230 | 3h_1% | MT81_63 |
| bt_0231 | 3h_1% | MT81_63 |
| bt_0232 | 3h_1% | MT81_63 |
| bt_0233 | 3h_1% | MT81_63 |
| bt_0234 | 3h_1% | MT81_63 |
| bt_0235 | 3h_1% | MT81_63 |
| bt_0236 | 3h_1% | MT81_63 |
| bt_0409 | 3h_1% | MT82_23 |
| bt_0410 | 3h_1% | MT82_23 |
| bt_0720 | 3h_1% | MT82_23 |
| bt_0721 | 3h_1% | MT82_23 |
| bt_0722 | 3h_1% | MT82_23 |
| bt_0049 | 6h_0.5% | MT82_26 |
| bt_0050 | 6h_0.5% | MT82_26 |
| bt_0051 | 6h_0.5% | MT82_26 |
| bt_0052 | 6h_0.5% | MT82_26 |
| bt_0055 | 6h_0.5% | MT82_26 |
| bt_0056 | 6h_0.5% | MT82_26 |
| bt_0058 | 6h_0.5% | MT82_26 |
| bt_0059 | 6h_0.5% | MT82_26 |
| bt_0113 | 3h_1% | MT82_33 |
| bt_0114 | 3h_1% | MT82_33 |
| bt_0115 | 3h_1% | MT82_33 |
| bt_0116 | 3h_1% | MT82_33 |
| bt_0117 | 3h_1% | MT82_33 |
| bt_0118 | 3h_1% | MT82_33 |
| bt_0119 | 3h_1% | MT82_33 |
| bt_0120 | 3h_1% | MT82_33 |
| bt_0121 | 3h_1% | MT82_33 |
| bt_0122 | 3h_1% | MT82_33 |
| bt_0123 | 3h_1% | MT82_33 |
| bt_0124 | 3h_1% | MT82_33 |
| bt_0125 | 3h_1% | MT82_33 |
| bt_0126 | 3h_1% | MT82_33 |
| bt_0127 | 3h_1% | MT82_33 |
| bt_0217 | 3h_1% | MT82_33 |
| bt_0218 | 3h_1% | MT82_33 |
| bt_0219 | 3h_1% | MT82_33 |
| bt_0220 | 3h_1% | MT82_33 |
| bt_0221 | 3h_1% | MT82_33 |
| bt_0222 | 3h_1% | MT82_33 |
| bt_0223 | 3h_1% | MT82_33 |
| bt_0224 | 3h_1% | MT82_33 |
| bt_0225 | 3h_1% | MT82_33 |
| bt_0226 | 3h_1% | MT82_33 |
| bt_0227 | 3h_1% | MT82_33 |
| bt_0228 | 3h_1% | MT82_33 |
| bt_0229 | 3h_1% | MT82_33 |
| bt_0107 | 3h_1% | MT82_73 |
| bt_0108 | 3h_1% | MT82_73 |
| bt_0109 | 3h_1% | MT82_73 |
| bt_0110 | 3h_1% | MT82_73 |
| bt_0111 | 3h_1% | MT82_73 |
| bt_0112 | 3h_1% | MT82_73 |
| bt_0289 | 3h_1% | MT82_73 |
| bt_0293 | 3h_1% | MT82_73 |
| bt_0559 | 3h_1% | MT82_73 |
| bt_0560 | 3h_1% | MT82_73 |
| bt_0694 | 3h_1% | MT82_73 |
| bt_0695 | 3h_1% | MT82_73 |
| bt_0696 | 3h_1% | MT82_73 |
| bt_0862 | 48h_0.06% | MT84_14 |
| bt_0863 | 48h_0.06% | MT84_14 |
| bt_0864 | 48h_0.06% | MT84_14 |
| bt_0865 | 48h_0.06% | MT84_14 |
| bt_0866 | 48h_0.06% | MT84_14 |
| bt_0868 | 48h_0.06% | MT84_14 |
| bt_0870 | 48h_0.06% | MT84_14 |
| bt_0035 | 48h_0.06% | MT84_14 |
| bt_0036 | 48h_0.06% | MT84_14 |
| bt_0037 | 48h_0.06% | MT84_14 |
| bt_0038 | 48h_0.06% | MT84_14 |
| bt_0810 | 6h_0.5% | MT85_26 |
| bt_0811 | 6h_0.5% | MT85_26 |
| bt_0812 | 6h_0.5% | MT85_26 |
| bt_0815 | 6h_0.5% | MT85_26 |
| bt_0816 | 6h_0.5% | MT85_26 |
| bt_0446 | 6h_0.5% | MT86_26 |
| bt_0447 | 6h_0.5% | MT86_26 |
| bt_0448 | 6h_0.5% | MT86_26 |
| bt_0449 | 6h_0.5% | MT86_26 |
| bt_0450 | 6h_0.5% | MT86_26 |
| bt_0451 | 6h_0.5% | MT86_26 |
| bt_0452 | 6h_0.5% | MT86_26 |
| bt_0454 | 6h_0.5% | MT86_26 |
| bt_0455 | 6h_0.5% | MT86_26 |
| bt_0456 | 6h_0.5% | MT86_26 |
| bt_0716 | 3h_1% | MT86_73 |
| bt_0717 | 3h_1% | MT86_73 |
| bt_0771 | 3h_1% | MT86_73 |
| bt_0457 | 48h_0.06% | MT87_64 |
| bt_0458 | 48h_0.06% | MT87_64 |
| bt_0459 | 48h_0.06% | MT87_64 |
| bt_0460 | 48h_0.06% | MT87_64 |
| bt_0461 | 48h_0.06% | MT87_64 |
| bt_0464 | 48h_0.06% | MT87_64 |
| bt_0465 | 48h_0.06% | MT87_64 |
| bt_0466 | 48h_0.06% | MT87_64 |
| bt_0467 | 48h_0.06% | MT87_64 |
| bt_0468 | 48h_0.06% | MT87_64 |
| bt_0469 | 48h_0.06% | MT87_64 |
| bt_0470 | 48h_0.06% | MT87_64 |
| bt_0471 | 48h_0.06% | MT87_64 |
| bt_0472 | 48h_0.06% | MT87_64 |
| bt_0473 | 48h_0.06% | MT87_64 |
| bt_0474 | 48h_0.06% | MT87_64 |
| bt_0676 | 3h_1% | MT88_83 |
| bt_0677 | 3h_1% | MT88_83 |
| bt_0678 | 3h_1% | MT88_83 |
| bt_0679 | 3h_1% | MT88_83 |
| bt_0213 | 3h_1% | MT89_53 |
| bt_0214 | 3h_1% | MT89_53 |
| bt_0215 | 3h_1% | MT89_53 |
| bt_0216 | 3h_1% | MT89_53 |
| bt_0576 | 3h_1% | MT89_53 |
| bt_0577 | 3h_1% | MT89_53 |
| bt_0578 | 3h_1% | MT89_53 |
| bt_0579 | 3h_1% | MT89_53 |
| bt_0580 | 3h_1% | MT89_53 |
| bt_0581 | 3h_1% | MT89_53 |
| bt_0582 | 3h_1% | MT89_53 |
| bt_0583 | 3h_1% | MT89_53 |
| bt_0584 | 3h_1% | MT89_53 |
| bt_0608 | 6h_0.5% | MT89_76 |
| bt_0609 | 6h_0.5% | MT89_76 |
| bt_0610 | 6h_0.5% | MT89_76 |
| bt_0611 | 6h_0.5% | MT89_76 |
| bt_0612 | 6h_0.5% | MT89_76 |
| bt_0613 | 6h_0.5% | MT89_76 |
| bt_0614 | 6h_0.5% | MT89_76 |
| bt_0547 | 24h_0.125% | MT9_12 |
| bt_0548 | 24h_0.125% | MT9_12 |
| bt_0549 | 24h_0.125% | MT9_12 |
| bt_0627 | 48h_0.06% | MT9_94 |
| bt_0628 | 48h_0.06% | MT9_94 |
| bt_0629 | 48h_0.06% | MT9_94 |
| bt_0631 | 48h_0.06% | MT9_94 |
| bt_0632 | 48h_0.06% | MT9_94 |
| bt_0633 | 48h_0.06% | MT9_94 |
| bt_0634 | 48h_0.06% | MT9_94 |
| bt_0635 | 48h_0.06% | MT9_94 |
| bt_0636 | 48h_0.06% | MT9_94 |
| bt_0637 | 48h_0.06% | MT9_94 |
| bt_0638 | 48h_0.06% | MT9_94 |
| bt_0639 | 48h_0.06% | MT9_94 |
| bt_0640 | 48h_0.06% | MT9_94 |
| bt_0641 | 48h_0.06% | MT9_94 |
| bt_0177 | 3h_1% | MT90_23 |
| bt_0178 | 3h_1% | MT90_23 |
| bt_0179 | 3h_1% | MT90_23 |
| bt_0180 | 3h_1% | MT90_23 |
| bt_0181 | 3h_1% | MT90_23 |
| bt_0182 | 3h_1% | MT90_23 |
| bt_0183 | 3h_1% | MT90_23 |
| bt_0237 | 3h_1% | MT90_23 |
| bt_0238 | 3h_1% | MT90_23 |
| bt_0239 | 3h_1% | MT90_23 |
| bt_0240 | 3h_1% | MT90_23 |
| bt_0385 | 3h_1% | MT90_23 |
| bt_0388 | 3h_1% | MT90_23 |
| bt_0389 | 3h_1% | MT90_23 |
| bt_0297 | 3h_1% | MT90_83 |
| bt_0299 | 3h_1% | MT90_83 |
| bt_0300 | 3h_1% | MT90_83 |
| bt_0302 | 3h_1% | MT90_83 |
| bt_0303 | 3h_1% | MT90_83 |
| bt_0304 | 3h_1% | MT90_83 |
| bt_0305 | 3h_1% | MT90_83 |
| bt_0306 | 3h_1% | MT90_83 |
| bt_0307 | 3h_1% | MT90_83 |
| bt_0308 | 3h_1% | MT90_83 |
| bt_0309 | 3h_1% | MT90_83 |
| bt_0310 | 3h_1% | MT90_83 |
| bt_0311 | 3h_1% | MT90_83 |
| bt_0312 | 3h_1% | MT90_83 |
| bt_0313 | 3h_1% | MT90_83 |
| bt_0314 | 3h_1% | MT90_83 |
| bt_0315 | 3h_1% | MT90_83 |
| bt_0316 | 3h_1% | MT90_83 |
| bt_0317 | 3h_1% | MT90_83 |
| bt_0318 | 3h_1% | MT90_83 |
| bt_0319 | 3h_1% | MT90_83 |
| bt_0320 | 3h_1% | MT90_83 |
| bt_0321 | 3h_1% | MT90_83 |
| bt_0322 | 3h_1% | MT90_83 |
| bt_0323 | 3h_1% | MT90_83 |
| bt_0324 | 3h_1% | MT90_83 |
| bt_0325 | 3h_1% | MT90_83 |
| bt_0326 | 3h_1% | MT90_83 |
| bt_0327 | 3h_1% | MT90_83 |
| bt_0328 | 3h_1% | MT90_83 |
| bt_0329 | 3h_1% | MT90_83 |
| bt_0330 | 3h_1% | MT90_83 |
| bt_0331 | 3h_1% | MT90_83 |
| bt_0332 | 3h_1% | MT90_83 |
| bt_0333 | 3h_1% | MT90_83 |
| bt_0334 | 3h_1% | MT90_83 |
| bt_0335 | 3h_1% | MT90_83 |
| bt_0336 | 3h_1% | MT90_83 |
| bt_0337 | 3h_1% | MT90_83 |
| bt_0338 | 3h_1% | MT90_83 |
| bt_0339 | 3h_1% | MT90_83 |
| bt_0340 | 3h_1% | MT90_83 |
| bt_0341 | 3h_1% | MT90_83 |
| bt_0342 | 3h_1% | MT90_83 |
| bt_0343 | 3h_1% | MT90_83 |
| bt_0344 | 3h_1% | MT90_83 |
| bt_0345 | 3h_1% | MT90_83 |
| bt_0346 | 3h_1% | MT90_83 |
| bt_0347 | 3h_1% | MT90_83 |
| bt_0348 | 3h_1% | MT90_83 |
| bt_0349 | 3h_1% | MT90_83 |
| bt_0350 | 3h_1% | MT90_83 |
| bt_0351 | 3h_1% | MT90_83 |
| bt_0352 | 3h_1% | MT90_83 |
| bt_0353 | 3h_1% | MT90_83 |
| bt_0354 | 3h_1% | MT90_83 |
| bt_0355 | 3h_1% | MT90_83 |
| bt_0356 | 3h_1% | MT90_83 |
| bt_0357 | 3h_1% | MT90_83 |
| bt_0481 | 3h_1% | MT90_83 |
| bt_0482 | 3h_1% | MT90_83 |
| bt_0483 | 3h_1% | MT90_83 |
| bt_0484 | 3h_1% | MT90_83 |
| bt_0485 | 3h_1% | MT90_83 |
| bt_0486 | 3h_1% | MT90_83 |
| bt_0487 | 3h_1% | MT90_83 |
| bt_0488 | 3h_1% | MT90_83 |
| bt_0489 | 3h_1% | MT90_83 |
| bt_0490 | 3h_1% | MT90_83 |
| bt_0491 | 3h_1% | MT90_83 |
| bt_0492 | 3h_1% | MT90_83 |
| bt_0493 | 3h_1% | MT90_83 |
| bt_0592 | 3h_1% | MT90_83 |
| bt_0593 | 3h_1% | MT90_83 |
| bt_0595 | 3h_1% | MT90_83 |
| bt_0596 | 3h_1% | MT90_83 |
| bt_0597 | 3h_1% | MT90_83 |
| bt_0930 | 3h_1% | MT90_83 |
| bt_0932 | 3h_1% | MT90_83 |
| bt_0564 | 3h_1% | MT91_23 |
| bt_0565 | 3h_1% | MT91_23 |
| bt_0566 | 3h_1% | MT91_23 |
| bt_0567 | 3h_1% | MT91_23 |
| bt_0568 | 3h_1% | MT91_23 |
| bt_0569 | 3h_1% | MT91_23 |
| bt_0570 | 3h_1% | MT91_23 |
| bt_0797 | 6h_0.5% | MT91_66 |
| bt_0664 | 3h_1% | MT92_63 |
| bt_0665 | 3h_1% | MT92_63 |
| bt_0791 | 3h_1% | MT92_63 |
| bt_0793 | 3h_1% | MT92_63 |
| bt_0794 | 3h_1% | MT92_63 |
| bt_0405 | 48h_0.06% | MT93_14 |
| bt_0406 | 48h_0.06% | MT93_14 |
| bt_0407 | 48h_0.06% | MT93_14 |
| bt_0408 | 48h_0.06% | MT93_14 |
| bt_0669 | 6h_0.5% | MT93_26 |
| bt_0511 | 3h_1% | MT93_43 |
| bt_0512 | 3h_1% | MT93_43 |
| bt_0514 | 3h_1% | MT93_43 |
| bt_0515 | 3h_1% | MT93_43 |
| bt_0516 | 3h_1% | MT93_43 |
| bt_0514b | 3h_1% | MT93_43 |
| bt_0515b | 3h_1% | MT93_43 |
| bt_0516b | 3h_1% | MT93_43 |
| bt_0517 | 3h_1% | MT93_43 |
| bt_0660 | 3h_1% | MT93_43 |
| bt_0661 | 3h_1% | MT93_43 |
| bt_0662 | 3h_1% | MT93_43 |
| bt_0663 | 3h_1% | MT93_43 |
| bt_0763 | 3h_1% | MT93_53 |
| bt_0475 | 3h_1% | MT94_33 |
| bt_0476 | 3h_1% | MT94_33 |
| bt_0477 | 3h_1% | MT94_33 |
| bt_0478 | 3h_1% | MT94_33 |
| bt_0479 | 3h_1% | MT94_33 |
| bt_0480 | 3h_1% | MT94_33 |
| bt_0494 | 3h_1% | MT94_33 |
| bt_0495 | 3h_1% | MT94_33 |
| bt_0496 | 3h_1% | MT94_33 |
| bt_0497 | 3h_1% | MT94_33 |
| bt_0498 | 3h_1% | MT94_33 |
| bt_0499 | 3h_1% | MT94_33 |
| bt_0598 | 3h_1% | MT94_33 |
| bt_0599 | 3h_1% | MT94_33 |
| bt_0600 | 3h_1% | MT94_33 |
| bt_0602 | 3h_1% | MT94_33 |
| bt_0603 | 3h_1% | MT94_33 |
| bt_0604 | 3h_1% | MT94_33 |
| bt_0281 | 3h_1% | MT94_43 |
| bt_0282 | 3h_1% | MT94_43 |
| bt_0283 | 3h_1% | MT94_43 |
| bt_0284 | 3h_1% | MT94_43 |
| bt_0285 | 3h_1% | MT94_43 |
| bt_0286 | 3h_1% | MT94_43 |
| bt_0287 | 3h_1% | MT94_43 |
| bt_0039 | 3h_1% | MT94_43 |
| bt_0040 | 3h_1% | MT94_43 |
| bt_0041 | 3h_1% | MT94_43 |
| bt_0042 | 3h_1% | MT94_43 |
| bt_0043 | 3h_1% | MT94_43 |
| bt_0044 | 3h_1% | MT94_43 |
| bt_0045 | 3h_1% | MT94_43 |
| bt_0046 | 3h_1% | MT94_43 |
| bt_0047 | 3h_1% | MT94_43 |
| bt_0048 | 3h_1% | MT94_43 |
| bt_0200 | 6h_0.5% | MT94_56 |
| bt_0201 | 6h_0.5% | MT94_56 |
| bt_0202 | 6h_0.5% | MT94_56 |
| bt_0203 | 6h_0.5% | MT94_56 |
| bt_0728 | 3h_1% | MT94_63 |
| bt_0729 | 3h_1% | MT94_63 |
| bt_0730 | 3h_1% | MT94_63 |
| bt_0731 | 3h_1% | MT94_63 |
| bt_0433 | 48h_0.06% | MT94_64 |
| bt_0434 | 48h_0.06% | MT94_64 |
| bt_0838 | 6h_0.5% | MT95_26 |
| bt_0840 | 6h_0.5% | MT95_26 |
| bt_0857 | 6h_0.5% | MT95_66 |
| bt_0642 | 3h_1% | MT97_93 |
| bt_0643 | 3h_1% | MT97_93 |
| bt_0644 | 3h_1% | MT97_93 |
| bt_0647 | 3h_1% | MT97_93 |
| bt_0836 | 3h_1% | MT97_93 |
| bt_0837 | 3h_1% | MT97_93 |
| bt_0163 | 3h_1% | MT98_13 |
| bt_0164 | 3h_1% | MT98_13 |
| bt_0165 | 3h_1% | MT98_13 |
| bt_0762 | 3h_1% | MT98_13 |
| bt_0500 | 3h_1% | MT99_53 |
| bt_0501 | 3h_1% | MT99_53 |
| bt_0502 | 3h_1% | MT99_53 |
| bt_0503 | 3h_1% | MT99_53 |
| bt_0504 | 3h_1% | MT99_53 |
| bt_0505 | 3h_1% | MT99_53 |
| bt_0506 | 3h_1% | MT99_53 |
| bt_0507 | 3h_1% | MT99_53 |
| bt_0508 | 3h_1% | MT99_53 |
| bt_0509 | 3h_1% | MT99_53 |
| bt_0510 | 3h_1% | MT99_53 |
| bt_0430 | 3h_1% | MT99_63 |
| bt_0431 | 3h_1% | MT99_63 |
| bt_0432 | 3h_1% | MT99_63 |
| bt_0572 | 3h_1% | MT99_83 |
| bt_0573 | 3h_1% | MT99_83 |
