# Supplementary material for: Induction, rapid fixation and retention of mutations in vegetatively propagated banana
Source: Plant Biotechnol J. 2012 Dec;10(9):1056–66. doi: 10.1111/j.1467-7652.2012.00733.x (PMC3533788; doi:10.1111/j.1467-7652.2012.00733.x)
Supplement: Supplementary file 3 [file pbi0010-1056-SD3.doc]

| **Table S3** Expected and observed mutations for tested amplicons | | | | | | |  |
| --- | --- | --- | --- | --- | --- | --- | --- |
|  |  |  |  |  |  |  |  |
| A. Percentage of expected alleles calculated using CODDLE | | | | |  |  |  |
| **Gene Target** | **Target size** | **Truncation changes (%)** | **Missense Changes (%)** | **Nonsilent Changes (%)** | **Truncation (#)** | **Missense (#)** |  |
| ACETRANS | 1415.00 | 0.06 | 0.54 | 0.59 | 83.49 | 757.03 |  |
| AMTHLTR | 1410.00 | 0.01 | 0.32 | 0.33 | 18.33 | 446.97 |  |
| DNAJ | 1395.00 | 0.08 | 0.66 | 0.74 | 110.21 | 923.49 |  |
| ELF3 | 1416.00 | 0.05 | 0.54 | 0.59 | 65.14 | 764.64 |  |
| FTSJMT | 1369.00 | 0.05 | 0.55 | 0.60 | 67.08 | 754.32 |  |
| GHF17 | 1402.00 | 0.03 | 0.59 | 0.62 | 44.86 | 827.18 |  |
| MALSYN | 1402.00 | 0.04 | 0.42 | 0.46 | 57.48 | 583.23 |  |
| NPH3 | 1427.00 | 0.06 | 0.58 | 0.65 | 91.33 | 831.94 |  |
| PAAL2 | 1422.00 | 0.03 | 0.48 | 0.50 | 39.82 | 678.29 |  |
| PUF | 1472.00 | 0.03 | 0.60 | 0.63 | 48.58 | 878.78 |  |
| RNDR | 1500.00 | 0.06 | 0.42 | 0.48 | 91.50 | 622.50 |  |
|  | 15630.00 |  |  |  | 717.80 | 8068.38 |  |
|  |  |  |  |  |  |  |  |
| **% of total** |  |  |  |  | **0.05** | **0.52** |  |
|  |  |  |  |  | **4.59** | **51.62** |  |
|  |  |  |  |  |  |  |  |
| **% Silent ^a^** | **43.79** |  |  |  |  |  |  |
|  |  |  |  |  |  |  |  |
|  |  |  |  |  |  |  |  |
| B. Observed versus expected alleles | | | |  |  |  |  |
|  | **Missense** |  | **Truncation** |  | **Silent** |  |  |
|  | observed | expected | observed | expected | observed | expected |  |
|  | 16.00 | 17.03 | 5.00 | 1.52 | 12.00 | 14.45 |  |
|  |  |  |  |  |  |  |  |
| **%** | **48.48** |  | **15.15** |  | **36.36** |  |  |
|  |  |  |  |  |  |  |  |
| **Binomial distribution** | **0.0002** |  |  |  |  |  |  |
|  |  |  |  |  |  |  |  |
|  |  |  |  |  |  |  |  |
| ^a^ Percentage of expected alleles not being truncation or missense | | | | | |  |  |
|  |  |  |  |  |  |  |  |
|  |  |  |  |  |  |  |  |
