# Supplementary material for: Induction, rapid fixation and retention of mutations in vegetatively propagated banana
Source: Plant Biotechnol J. 2012 Dec;10(9):1056–66. doi: 10.1111/j.1467-7652.2012.00733.x (PMC3533788; doi:10.1111/j.1467-7652.2012.00733.x)
Supplement: Supplementary file 4 [file pbi0010-1056-SD4.doc]

| **Table S4** Frequencies of observed phenotypes after 24 months greenhouse growth | | | | |  |
| --- | --- | --- | --- | --- | --- |
| **Line** | **Plants after 24 months (#)** | **Phenotype** | **Plants with phenotype after 24 months (#)** | **Phenotype after 24 months (%)** |  |
|  |  |  |  |  |  |
| MT1_24 | 2 | Leaf discoloration | 2 | 100 |  |
| MT22_26 | 2 | Wild-type | 1 | 50 |  |
|  |  | Leaf discoloration | 1 | 50 |  |
| MT3_33 | 6 | Wild-type | 1 | 17 |  |
|  |  | Leaf discoloration | 5 | 83 |  |
| MT33_63 | 12 | Yellow streaks^a^ | 12 | 100 |  |
| MT4_52 | 1 | Light leaf discoloration | 1 | 100 |  |
| MT41_43 | 1 | Light leaf discoloration | 1 | 100 |  |
| MT41_73 | 1 | Very narrowed and long leaves | 1 | 100 |  |
| MT43_5 | 5 | Dark green vein/leaves light green | 5 | 100 |  |
| MT47_33 | 3 | Yellow streaks | 3 | 100 |  |
| Mt49_44 | 1 | Narrow leaves/light yellow veins | 1 | 100 |  |
| MT50_92 | 1 | Wild-type | 1 | 100 |  |
| MT52_34 | 2 | Wild-type | 2 | 100 |  |
| MT54_74 | 3 | Narrowed leaves/Wavy edge and curly vein | 3 | 100 |  |
| MT57_2 | 2 | Rolled leaves | 2 | 100 |  |
| MT62_5 | 1 | Light leaf discoloration | 1 | 100 |  |
| MT62_73 | 1 | Dark/strait green vein | 1 | 100 |  |
| MT79_43 | 1 | Wild-type | 1 | 100 |  |
| MT80_53 | 6 | Yellow streaks in the lamina of the leaves | 6 | 100 |  |
| MT86_26 | 1 | Leaves narrowing towards the top of the leaf/green vein with light discoloration | 1 | 100 |  |
| MT87_64 | 1 | Wild-type | 1 | 100 |  |
| MT89_76 | 6 | Wild-type | 1 | 17 |  |
|  |  | Leaf discoloration | 3 | 50 |  |
|  |  | Narrowed leaves | 2 | 33 |  |
| MT90_23 | 5 | Young leaves with partial yellow streaks, when older normal | 5 | 100 |  |
| MT90_83 | 28 | Narrow Leaf | 26 | 93 |  |
| MT91_23 | 3 | Young leaves yellow | 1 | 33 |  |
|  |  | Narrowed leaves | 2 | 67 |  |
| MT93_43 | 6 | Leaf discoloration/dark green vein | 3 | 50 |  |
|  |  | Shorter leaves | 3 | 50 |  |
| MT94_33 | 7 | Dark green vein with yellow in between | 7 | 100 |  |
| MT98_13 | 1 | Green vein/Yellow leaf discoloration | 1 | 100 |  |
|  |  |  |  |  |  |
| **Total** | **109** |  |  |  |  |
| **Total Phenotype** | |  | **99** | **90.83** |  |
|  |  |  |  |  |  |
| ^a^ Young leaves/plants very yellow, older leaves are changing to be darker | | | |  |  |
|  |  |  |  |  |  |
|  |  |  |  |  |  |
